# Ten simple rules to leverage large language models for getting grants

**DOI:** 10.1371/journal.pcbi.1011863

**Published:** 2024-03-01

**Authors:** Elizabeth Seckel, Brandi Y. Stephens, Fatima Rodriguez

**Affiliations:** Division of Cardiovascular Medicine, Stanford University School of Medicine, Stanford, California, United States of America; Carnegie Mellon University, UNITED STATES

## Introduction

The recent leap in performance of large language models (LLMs), a subclass of artificial intelligence (AI) algorithms that includes OpenAI’s ChatGPT, Google Bard, and Microsoft’s Copilot (formerly Bing Chat), ushered a revolution in artificial text generation. These systems, trained on billions of documents, are sophisticated enough to fool human users into thinking they are conversing with other humans [1,2].

In academia, LLM-driven chatbots have become popular tools to help draft and revise scientific text [3,4], with some going as far as including them as coauthors [5]. Enthusiasts highlight the ability of these systems to summarize entire articles, simplify jargon-laden paragraphs, and improve the clarity and conciseness of drafts, particularly for non-native English writers [6–8]. On the other hand, others have advocated for strict boundaries and restrictions [5,9,10], citing ethical and privacy concerns as well as the tendency of these tools to “hallucinate”—or confabulate and fabricate—facts and references [11]. LLMs are fed enormous amounts of information and use statistics to predict the next word in a sentence [12]. By doing so, they generate grammatically and semantically correct text in response to prompts but are unable to estimate the uncertainty or truth of their predictions—resulting in hallucinations. This also means that the generated text can be borrowed *verbatim* from existing sources, which has led to a growing number of copyright lawsuits [13,14].

**Fig 1:**
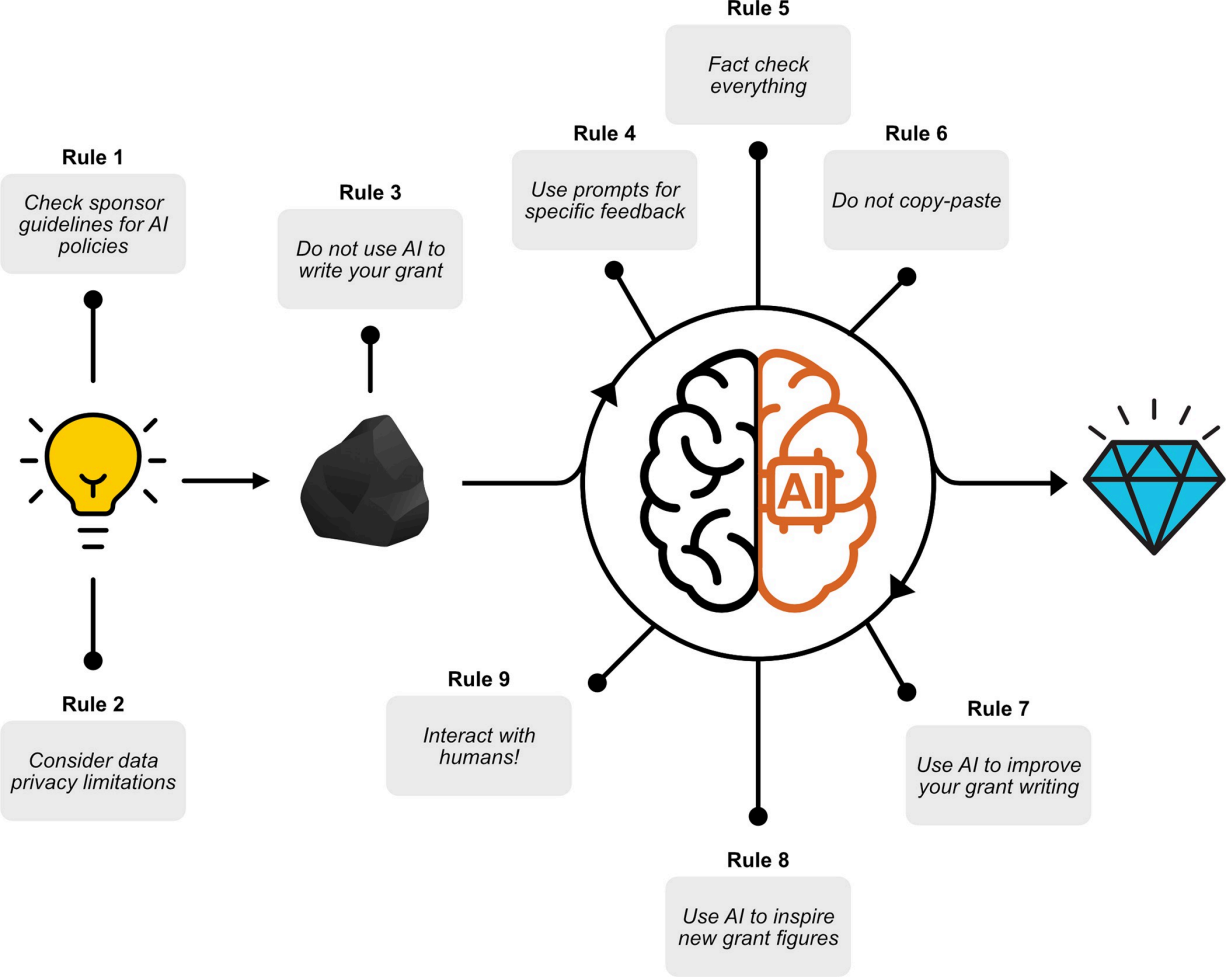
Ten rules for leveraging LLMs for getting grants. Proposal development timeline to illustrate at what point in your grant writing journey to incorporate each rule. Light bulb and coal icons used in Fig 1 were adapted from https://www.svgrepo.com/svg/524676/lightbulb-minimalistic and https://www.svgrepo.com/svg/398225/rock, respectively.

As writers of scientific proposals, we believe that writing proposals is a very personal exercise where the final product is best when imbued with the ideas, style, and personality of the writer. The iterative process of drafting and refining also helps develop scientific writing skills [15], which are essential for a successful long-term career in academia. We also believe, however, that scientists can benefit immensely from including AI in this process, as assistants or makeshift reviewers, in particular as the algorithms that power these systems become better and more widely available. This article aims to strike a delicate balance—an enthusiastic yet cautionary tale outlining 10 best practice tips (summarized in Fig 1) for using LLMs during your grant writing journey.

## Rule 1: Check the guidelines of the funding agency regarding AI

Several publishers and funding agencies have issued specific—but diverse—guidelines regarding the use of AI chatbots in publications and grant applications. While publishers such as *Science* initially took a very restrictive stance, equating using AI to plagiarism and forbidding its use in any submissions to its journals [16], many now simply forbid chatbots from being listed as authors but allow their use in publications if properly acknowledged [9,17]. Going a step further, Springer Nature has recently released Curie, a new AI-powered writing assistant for academic researchers, especially for those whose first language is not English [18]. For grants, the American Heart Association allows writers to use AI freely as long as they disclose it at the time of submission [19]. The National Science Foundation encourages submitters to indicate if and how generative AI technology was used to develop their proposals while cautioning that this technology can introduce fabrication, falsification, or plagiarism, which would constitute research misconduct [20]. The National Institutes of Health, on the other hand, do “not know, or ask, who wrote an application” but warns that scientists using AI tools to help write applications do so at their own risk due to automated systems checking for plagiarism or false information [21]. As more and more institutions draft their own guidelines regarding AI usage, and given that existing policies are likely to change over time, it is imperative that you continuously check the rules for the funding agency where you are submitting your grant. Whether the funder requests this or not, we recommend disclosing any AI usage in your grant applications.

## Rule 2: Consider data privacy limitations

AI chatbots improve by learning from interactions with their users. Indeed, all the publicly available AI chatbots, including ChatGPT and Google Bard, save your prompts and conversations with the explicit goal of improving their algorithms. For example, although ChatGPT offers a way to delete your data, the company still advises users to refrain from sharing sensitive information with the AI. We argue that your grant application is, from draft to submission, extremely sensitive information that you would not want to share freely with a conversational AI. Your ideas and approach could be suggested to another user—a competitor!—in a future iteration of the chatbot. To this end, we urge you to weigh the benefits of using *publicly* available AI chatbots to help you with your grant application. Always check the data storage settings before using any of the chatbots. Finally, if available at your institution, or you have the computational resources, a self-hosted LLM restricted to offline access may offer improved security and privacy, although at a cost of accuracy and/or performance.

## Rule 3: Don’t use AI to write your grant

A good rule of thumb is the first draft of any section must come from you. Because of how these LLMs are trained, their output is not guaranteed to be original or scientifically valid. So, while it can be tempting to ask the LLM to generate an initial draft for you starting from an idea or a couple of sentences, avoiding the dreaded “blank page,” we do not recommend engaging with the AI until you have a draft and are ready to start the revision stage of the writing process. This advice applies both to the entire grant and to individual sections. Ultimately, your grant must reflect you as a scientist—your scientific ideas, your preliminary data, and your novel approach, described in your own words.

## Rule 4: Use custom prompts for specific feedback

Just as when requesting feedback from your human colleagues and mentors, the more specific you are in what and how you would like them to review, the better, more focused, and ultimately more helpful feedback you receive. Requesting feedback from an LLM is no different. In our experience, we found LLMs excel when provided with instructions to narrow down their focus to a specific task or section, which you can achieve by using custom prompts. Writing that you are a postdoctoral fellow applying for a career development award from the American Heart Association and that you would like feedback on how closely aligned your text is with their mission can help the LLM make better and more appropriate comments and adjustments to your text. Then, narrow down the focus of the LLM to each specific section, instead of dumping your entire application on the screen. You can then finish by asking it to evaluate if the different sections make up a coherent and cohesive story. Moreover, if you are a non-native English writer, you can ask the LLM to improve grammar and spelling in your proposal and lessen the burden of writing in a foreign language. Finally, make use of specific prompt features of each LLM; for example, ChatGPT allows you to use custom prompts to set rules for the LLM to use in all its subsequent answers, helping you get more coherent and specific feedback [22]. We include these and other ideas for specific prompts (generated by us) in Table 1 below to help get you started.

**Table 1:** Large language model sample prompt text.

**To enhance text clarity** “As a non-native English speaker, kindly help me revise the following text for improved understanding and clarity. Please check for spelling and sentence structure errors and suggest alternatives.” “What suggestions do you have to enhance the clarity of my text?” “Please identify any parts of my writing that may be difficult for a lay audience to understand.” **To make text more compelling** “Please provide feedback on my writing style and how I can make it more persuasive and compelling for the grant reviewer.” “I’m trying to hook my reader with a strong introduction. Can you suggest a more captivating first sentence to draw them in from the start?” **To improve structure and flow of text** “I want to improve the overall structure of my Specific Aims. What tips do you have to structure it more effectively?” **To better align with the funding agency’s mission** “I’m working on a postdoctoral fellowship application. Can you please review my closing paragraph and suggest ways to better align it with the American Heart Association’s mission?” **To better align with the review criteria** “I am applying to <<insert fellowship name>>. Please provide me feedback on how well I am addressing this review criteria: <<insert specific review criteria>>, and suggestions for what I am missing and how I can improve.”

## Rule 5: Fact check everything

Generative AI models such as LLMs are known to “hallucinate”—or fabricate—facts and references in response to prompts, given the nature of their training [11,23]. Although these models are steadily improving—ChatGPT 4.0 is reportedly 40% better at not hallucinating compared to previous versions [24]—there is still a non-negligible chance that they will produce outright fake information. For example, when writing this article, we asked the free and paid versions of ChatGPT to give us references for their best grant writing tips. The former returned a completely fake reference whose DOI resolved to a publication on *C*. *elegans* development, while the latter cited an earlier publication in *PLoS Comp Bio* on grant writing, but added a DOI to a paper on computational models of cerebellar Purkinje cells. Others have had similar experiences: In several studies evaluating the ability of LLMs to provide accurate references, the large majority of them were incorrect, and an alarming number were fabricated [23,25–27]. Following the advice in the previous rule, we suggest incorporating a statement in your custom instructions similar to the following from Twitter/X user @MushtaqBilalPhD: “You will respond like an academic colleague, citing claims, opinions, and figures from authentic, published sources. Avoid inventing sources, and if uncertain, acknowledge so.” [28]. Nonetheless, the bottom line is, *always fact check*, regardless of how convincing the AI writes.

## Rule 6: Don’t copy-paste; use the AI-generated text as inspiration

LLMs work by simply predicting what the next word should be. As such, they cannot interpret or understand content. However, this does not stop them from sounding very convincing. Besides hallucinating fake information, AIs can and will plagiarize existing text and will often contain biases—e.g., racial and gender bias—due to the nature of their training datasets [29,30]. On a less harmful note, LLMs tend to generate “fluff” unless specifically asked to be concise. For these reasons and those listed in Rules 3 and 5, you should not simply copy-paste text directly from the LLM into your grant application. Instead, exercise your critical thinking skills and read through the text carefully, using it as inspiration to make strategic edits to your own draft (see Rule 7 below). This step-by-step approach also helps you maintain control over the text and be on the lookout for potential unwanted changes to your application.

We do note, however, that websites like ChatGPT or Google Bard do not yet support text comparison (i.e., track changes) so it can be difficult to ascertain exactly what *did* change. One current workaround is to use the *Compare Documents* feature of Word or Google Docs and create a third document to highlight the differences before and after LLM editing.

## Rule 7: Use this iterative process to become a better grant writer

Humans and AIs alike learn by consuming data and iterating. After every response from the AI, go over each suggested change and ask yourself: Would this change strengthens my application? Is this phrasing really easier to understand? Was it consistent in the changes it applied throughout the text, for example, by using mostly active voice instead of passive voice? In this way, much like the AIs, you can detect patterns in the suggestions and supervise your own “reinforcement learning.” By iterating back and forth with the AI, you will learn to express your ideas more clearly and concisely, as well as to anticipate, respond to, and incorporate feedback, all essential skills for a long-term career in science [15]. Consider other ways that AIs are trained—how can you leverage these techniques to improve your grant writing skills further? For example, perhaps you can develop your own “training sets” with successful examples of the grant mechanism you are targeting. In reviewing these examples, do you notice patterns in what kind of text or ideas get funded?

## Rule 8: Use the AI for inspiration in developing figures

Generative AI tools are not restricted to text and words. Tools such as DALL-E-3 or Midjourney can create images based on text prompts. For example, you can describe a particular diagram or image you have in mind and use the result as inspiration for an actual figure in your grant. Alternatively, you can provide one of your own images and ask the AI how it interprets it, or if a specific aspect you wanted to emphasize is clear. Nonetheless, Rules 5 and 6 always apply; much like text generators, image generators suffer from issues with plagiarism and scientific correctness. We also refer the reader back to Rule 1: if using AI to help with figure generation, ensure this is permitted by the funding agency. Finally, while most of the most popular image generators come at a cost, some offer free trials that are ideal for experimenting.

## Rule 9: Don’t forget to interact with humans

While it can be exhilarating to have a lightning-fast assistant at your fingertips, always remember that no AI is a substitute for expert human review. It remains crucial to receive feedback from real humans—your peers and/or your mentors—during the many steps of your grant writing journey [31–33]. These individuals are much better equipped to pick up scientific and technical errors that otherwise might only be caught during the review process. This advice is especially important for junior scientists newer to grant writing. Requesting feedback, reviewing it carefully, and incorporating it in your draft is critical for your development as a scientist and in the creation of a strong and competitive proposal.

## Rule 10: Play!

The best way to learn how to use AI for grant writing is by playing and tinkering with it. Both OpenAI’s ChatGPT 3.5 and Google’s Bard, perhaps the most famous among many other generative AIs, allow you to create accounts and interact with the LLMs at no cost. Keeping in mind the advice in Rule 2, tinker with the prompts that you feed the AI and learn which work best. Experiment using different custom prompts, asking the same question in slightly different ways or to different chatbots. Ask questions for which you know the answer, to test the limitations of these generative models and learn how to fact check their responses. After all, generative models are here to stay and the sooner you get acquainted with their advantages and disadvantages, the faster you can unlock their potential to help you improve your grant applications and other scientific writing.

In conclusion, we hope this article has achieved the appropriate balance of caution and enthusiasm. By following these 10 simple rules, you can help avoid what we worry most about—having your AI-generated grant administratively rejected for plagiarism or your precious grant text incorporated into data training sets and suggested to your competitors asking similar prompts in the future. On a more positive note, we believe this technology has tremendous potential and are eager to see it better democratize the grant writing process—providing no (or low) cost grant writing aids to those of us without full-time grant writers at our beck and call, and helping non-native English writers overcome language barriers that are detrimental to equity in science [7,34].

To help kick-start this democratization, we have created a GitHub repository to collate and curate resources on this topic—https://github.com/eseckel/ai-for-grant-writing/—and we invite you to browse and contribute as you work on your next grant submission.
